# 

**Published:** 1959-07

**Authors:** 


MEDICAL JOURNAL OF THE SOUTH-WEST

(Incorporating the Bristol Medico-Chirurgical Journal)

Established 1883

VOL. 74 (iii) July 1959 NO. 273

### Contents

DECAY OF THE TEETH . . . . . *A. I. Darling* 53

LUNG CANCER—A SYMPOSIUM . . . *Dr. J. E. G. Pearson* 61

ALVEOLAR CELL CARCINOMA OF LUNG *Clinical Pathological Conference* 70

NEWS FROM SOCIETIES . . . . . . . . 74

NOTES AND NEWS . . . . . . . 77

APPOINTMENTS . . . . . . . . . 78

*PUBLISHED QUARTERLY*

*ANNUAL SUBSCRIPTION* 16/- *POST FREE*

PUBLISHED UNDER THE AUSPICES OF THE
BRISTOL MEDICO-CHIRURGICAL SOCIETY

### *Editorial Committee*

Dr. J. E. CATES, Department of Medicine, Bristol Royal Infirmary. *Editor.*
Dr. N. J. BROWN, Southmead Hospital, Bristol. *Assistant Editor.*
Dr. J. MACRAE, Ham Green Hospital.
Dr. R. C. WOFINDEN, Central Clinic, Bristol.
Mr. A. E. S. ROBERTS, Medical Library, University of Bristol. *Secretary.*

### *Local Correspondents*

*Bath* . . Mr. A. D. BATEMAN, 3 The Circus, Bath.
*Exeter* . . Dr. L. N. JACKSON, Mount Jocelyn, Crediton, Devon.
*Gloucester* . Dr. R. F. JARRETT, 9 College Green, Gloucester.
*Plymouth* . Dr. C. R. CROFT, Lexden, Hartley Av., Plymouth.
*Taunton* . Dr. M. MCALLEY, 1 The Crescent, Taunton.
*Torquay* . Dr. A. ROBINSON THOMAS, 20 Devon Square, Newton Abbot, Devon.
*Truro* . . Dr. F. D. M. HOCKING, St. Andrews, Perranporth, Cornwall.
*Yeovil* . . Mr. J. A. VERE NICOLL, Knapp House, Yeovil, Somerset.

### NOTICE TO CONTRIBUTORS

Original articles are invited, provided they have not been published elsewhere. Notes of forthcoming events, meetings held, degrees conferred, staff appointments, and other local news are welcomed. The editorial committee reserves the right to make literary corrections.

Illustrations should always be supplied ready for reproduction. All re-touchings or other operations required will be charged to the author. Line drawings should be drawn in Indian ink. We regret that we must ask authors to pay for making blocks, if this is necessary.

J. W. ARROWSMITH LTD., WINTERSTOKE ROAD,
BRISTOL.

### Notes and News

Mr. R. V. Cooke has been elected a member of the council of the Royal College of Surgeons of England.

Dr. Dorothy Shotton has been elected a member of the council of the Royal College of Obstetricians and Gynaecologists.

Dr. Allan Rogers, physiologist to the Trans-Antarctic expedition, has been presented with the triennial gold medal of the West London Medico-Chirurgical Society.

Dr. R. H. Parry has been appointed a member of the Local Government Commission for England under the Local Government Act 1958.

*F.R.C.P.:* John Apley, J. R. Bolton, J. J. Kempton.

*F.R.C.O.G.:* B. E. Blair.

### WESSEX RAHERE CLUB

The Autumn Dinner will take place at Lansdown Grove Hotel, Bath, on Saturday, 31st October, 1959. Further details can be obtained from Hon. Secretary, Mr. A. Daunt Bateman, 11 The Circus, Bath.

### EXAMINATION RESULTS

### *UNIVERSITY OF BRISTOL*

*Ph.D.*

C. C. Berry

*D.P.H.*

Febry, G. N.

Gryspeerdt, M. J.

Kinnear, E.

Lumb, Kathleen M.

Nairn, G. J.

Ryan, R. P.

Wallace, Barbara A.

*M.B., Ch.B.*

WITH SECOND CLASS HONOURS

Clegg, Patricia C.
Golding, P. R.
Harries, Sara J.
Knapp, M. S.
Smith, Sylvia J.

PASS

Bracey, Anne F. M.
Carr, R. M.
Cooper, A. J.
Cooper, J.
Dada, Mollie
Davies, J. W.
De Garis, S. T.
Etuk, E. S.
Goffin, R. B.
Hakes, J. A. A.
Howard, Valerie J.
Lipscomb, C. F.
Lovell, Patricia M.
Magbagbeola, J. A. O.
Magnus, R. V.
Medway, T. A.
Nash, A. G.
Odutola, Oyinade O.
Panahy, C.
Pilton, D. W.
Stant, Patricia
Steiner, H.
Sykes, D. W.
Symes, M. O.
Voyce, M. A.
Wale, D. H.
Walker, Anna G.
Walker, I. R.
White, A. G.
Wilson, A. M.

### PRIZES

*Foyle Prize:* Miss P. A. Clayton.

*Mackie Pathology Prize:* A. M. Wilson.

*Edward Fawcett Memorial Prize:* R. J. Paget.

*Thomas Francis Edgeworth and Francis Henry Edgeworth Prize:* C. E. Polkey.

*Tibbits Memorial Prize:* Miss A. G. Walker.

*David Rayner Prize in Gynaecology:* Dr. M. O. Symes.

*Gold Medal in Child Health and Paediatrics:* Dr. P. R. Golding.

*Green-Armytage Prize in Obstetrics and Gynaecology:* Miss S. J. Harries.

### *Appointments*

R. F. Woolmer, director of the Department of Anaesthetics at the Royal College of Surgeons, has been appointed the first British Oxygen professor of anaesthesia by the College Council.

Flavia Z. L. B. Cooper, assistant anaesthetist, Royal Samaritan Hospital for Women, Glasgow.

M. J. Dunn, assistant M.O./school M.O., North Devon area.

R. Daphne R. Sasieni, Deputy M.O.H., Benfleet, Canvey Island, Rayleigh and Rochford.

L. P. Spence, pathologist (grade A), health department, Trinidad.

A. M. Bain, surgical registrar, West Suffolk General Hospital.

### *UNITED BRISTOL HOSPITALS*
### *MARCH TO MAY* 1959

| *Name* | *Appointment* | *Formerly* |
|---|---|---|
| BRADFORD, Professor E. W. | Consultant Dental Surgeon (during tenure of the Chair of Dnetal Surgery in the University of Bristol). | Senior Lecturer in Preventive Dentistry & Parodontal Diseases in the University of St. Andrews. |
| JOHN, H. T., F.R.C.S. | Senior Surgical Registrar. | Senior Surgical Registrar, Royal Devon & Exeter Hospitals. |
| CROW, R. S., M.R.C.P. | Senior Medical Registrar. | Senior Medical Registrar, Royal Devon & Exeter Hospitals. |
| BOWDEN, B. J., D.O. | Senior Registrar in Ophthalmology. | Locum Outpatient Officer, Royal Free Hospital. |
| ADAMS, M. J. T., D.M.R.T. | Senior Registrar in Radiotherapy. | Registrar, Department of Radiotherapy, Middlesex Hospital. |
| WOOD, F. J. Y., M.D. (CANTAB). | Senior Registrar in Medicine (Cardiology). | Senior Medical Registrar, St. Thomas' Hospital. |
| MITCHELL, R. G., M.R.C.P.E. | Senior Registrar in Pathology. | Registrar, Department of Bacteriology, Newcastle. |
| WOODS, B., M.R.C.P. | Registrar in Dermatology. | S.H.O., Dulwich Hospital. |
| DANOS, E., M.B. | Registrar in Orthopaedics. | Locum Orthopaedic Registrar, Hospital of St. Cross, Rugby. |
| CARTER, Miss S. J., M.R.C.P. | Register in Radiology (Diagnostic). | Half-time Registrar in Radiology, United Bristol Hospitals. |
| JACKMAN, M., M.B., CH.B.(Bristol). | Registrar in Radiology (Diagnostic). | Half-time Registrar in Radiology, United Bristol Hospitals. |
| CLAREMONT, P. F., M.B., B.S. (Sydney). | Registrar in Radiology (Diagnostic). | Registrar in Radiology, St. Peter's Hospital, Chertsey. |
| O'MAHONY, J. B., B.D.S.R.C. (Q.U. Belfast) | Registrar in Dental Surgery. | Dental House Surgeon, Royal United Hospitals, Bath. |

Dr. D. R. COLES, M.B., CH.B., M.D., M.R.C.P. has been accorded the honor ary status of Senior Registrar in the Teaching Hospital during the tenure of his appointment as Second Lecturer in Medicine in the University of Bristol.

### *SOUTH WESTERN REGIONAL HOSPITAL BOARD*
### *MARCH TO JUNE* 1959

### *BRISTOL*

| *Name* | *Appointment* | *Formerly* |
|---|---|---|
| VELENTINE, M., M.D., D.P.M. | Consultant Psychiatrist, Bristol Mental Hospital Group. | Professor of Neuropsychiatry, University of Shiraz, Iran. |
| NAMBUDRIPAD, K. N., B.SC., M.B., B.S. (Madras), F.R.S.C.E. | Registrar in Department of Neurological Surgery, Frenchay Hospital, Bristol. | S.H.O., Department of Neurological Surgery, Frenchay. |

| *Name* | *Appointment* | *Formerly* |
|---|---|---|
| BAM, C., M.B., CH.B. (Cape Town). | Registrar in Obstetrics and Gynaecology, Southmead Hospital, Bristol. | Registrar, King Edward VIII Hospital, Durban. |
| HILL, B. W., M.B., CH.B. (Bristol). | Clinical Assistant in Dermattology, Cossham Memorial Hospital. | Is in general practice in Bristol. |
| WETHERED, H. H., B.A., M.B., B.CH. (Oxford). | Clinical Assistant in Dermattology, Southmead Hospital. | Is in general practice in Bristol. |
| LUMB, G. N., M.B., B.S. (Lond.), F.R.C.S. (Eng.). | Senior Registrar in Genitourinary Surgery, Southmead Hospital. | Surgical Registrar, Bath Group of Hospitals. |
| CHORLEY, G. E., M.B., B.S. (Lond.), D.A. | Anaesthetic Registrar, Frenchay Hospital. | Locum Registrar, Anaesthetics, Frenchay Hospital. |
| BIRD, R. L., M.B., B.S. (Lond.). | Registrar in Psychiatry, Bristol Mental Hospitals. | S.H.O., Bristol Mental Hospitals. |
| FOSTER, A. R., B.SC., M.B., CH.B. (Leeds), D.P.M. | Senior Registrar in Psychiatry, Bristol Mental Hospitals. | Registrar, Bristol Mental Hospital. |
| WESLEY-JAMES, O., M.B., B.S. (Lond.), F.R.C.S. (Edin. and Eng.). | Senior Surgical Registrar, Southmead Hospital. | Surgical Registrar, Radcliffe Infirmary, Oxford. |
| HANCOCK, D. O., M.B., B.S. (Lond.), F.R.C.S. (Edin. and Eng.). | Senior Registrar in Neurological Surgery, Frenchay Hospital | Registrar in Neurological Surgery, Frenchay Hospital. |
| LLOYD, A. T., M.B., CH.B. (Bristol). | Registrar in Psychiatry, Bristol Mental Hospitals. | R.M.O., The General Hospital, Otley, Yorks. |
| LESLIE, AGNES, M.B., B.S. (Madras), D.A. (Eng.). | Registrar in Anaesthetics, Southmead Hospital. | Resident Anaesthetist, Lister Hospital, Hitchin. |
| FORTUNE, D., M.B., CH.B. (Bristol). | Registrar in Pathology, Southmead Hospital. | S.H.O. in Pathology, Southmead Hospital. |
| DOLNY, L., B.M., B.CH., M.D. (Warsaw), D.P.M. | Assistant Psychiatrist, Bristol Mental Hospital Group. | Senior Psychiatric Registrar, Morgannwg Hospital, Bridgend. |
| WARREN, P. K. G., M.B., B.S. (Lond), M.R.C.P. (Lond.), D.P.M. | Assistant Psychiatrist, Bristol Mental Hospital Group. | Junior Hospital Medical Officer, Bristol Mental Hospital. |
| BRADFIELD, A. M., M.B., CH.B. (New Zealand), M.R.C.P. (Lond.), D.P.M. | Assistant Psychiatrist, Bristol Mental Hospital Group. | Senior Registrar, Barrow Hospital, Barrow Gurney. |

### *NORTH GLOUCESTERSHIRE*

| *Name* | *Appointment* | *Formerly* |
|---|---|---|
| HOOPER, A. J., M.B., B.CH. (Wales). | General Practitioner Anaesthetist, Dilke Memorial Hospital, Cinderford. | General Practice, Drybrook, Glos.; Locum G.P. Anaesthetist, Dilke Memorial Hospital. |
| SELSNICK, FRANCES, L.R.C.P. & S. (Edin.), L.R.F.P.S.G., F.R.C.S. (Eng.). | Registrar in General Surgery, Gloucester Royal Hospital. | S.H.O., City General Hospital, Stoke-on-Trent. |

### *BATH*

| *Name* | *Appointment* | *Formerly* |
|---|---|---|
| BOLT, G. L., M.B., B.S. (Lond.). | Registrar in General Medicine, Bath Group of Hospitals. | Locum Medical Registrar, St. Martins Hospital, Bath. |

| *Name* | *Appointment* | *Formerly* |
|---|---|---|
| BAZELEY, R. W., M.B., B.S. (Lond.). | Clinical Assistant in General Medicine in the Bath Group of Hospitals. | Is in general practice in Radstock. |
| BURROWES, W. L., M.D., B.CH. (Belfast), M.R.C.P. | Clinical Assistant in General Medicine in the Bath Group of Hospitals. | Is in general practice in Corsham. |
| ELLISON, D. J., M.B., CH.B. (Edin.), M.R.C.P. | Clinical Assistant in General Medicine in the Bath Group of Hospitals. | Is in general practice in Melksham. |
| PINCHING, J., B.M., B.CH. (Oxon), M.R.C.P., (Lond.). | Clinical Assistant in General Medicine in the Bath Group of Hospitals. | Is in general practice in Wells |
| DUCAT, E. F., M.B., CH.B. (Aberdeen). | Clinical Assistant in Paediatrics, R.U.H. | Is in general practice in Batheaston. |
| MUNRO, A. I., M.B., B.S. (Lond.) | Surgical Registrar, Bath Group of Hospitals. | Locum Registrar in general surgery, Bath Group of Hospitals. |
| KIRKUP, J. R., M.B., B.CHIR. (CANTAB), M.R.C.S. (Eng.), L.R.C.P. (Lond.). | Surgical Registrar, Bath Group of Hospitals. | S.H.O., Surgery, Manchester Royal Infirmary. |
| KILPATRICK, L. M.B., CH.B. (Edin.), D.(Obst.)R.C.O.G. | Re-appointed Registrar in Obstetrics and Gynaecology, Bath Group of Hospitals. | Registrar in Obstetrics and Gynaecology, Bath Group of Hospitals. |

### *DEVON AND CORNWALL*

| *Name* | *Appointment* | *Formerly* |
|---|---|---|
| JONES, I. S., M.A., F.R.C.S. | Consultant General Surgeon, Plymouth. | Senior Surgical Registrar, King's College Hospital. |
| HERON, W. C., M.B., CH.B., B.A.O. (Belfast), D.M.R.D. (Liverpool), D.M.R.D. (R.C.P. & S.), F.F.R. | Consultant Radiologist, Exeter. | Asst. Radiologist, Leicester R.I. |
| GRAINGER, C. N. | Clinical Assistant in Dermatology, Exeter. | Is in general practice in Totnes. |
| GUERRERO, F., M.B. (Santo Domingo), 1955. | Orthopaedic Registrar, Mount Gold Hospital, Plymouth. | Senior Resident in Surgery, Hotel Dieu Hospital, Kingston, Ont. |
| BARTLETT, L. H., B.A. (British Columbia), M.D., C.M. (McGill, Montreal), L.M.C.C. | Orthopaedic Registrar, Mount Gold Hospital, Plymouth. | Resident in Orthopaedic Surgery, Vancouver General Hospital. |
| PANDEY, M. R., M.B., B.S. (Calcutta), M.R.C.P. (Edin.). | Medical Registrar, South Devon and East Cornwall Hospital, Plymouth. | Clinical attachments and courses National Inst. of Neurology, National Heart Hospital, Brompton Hospital, Whittington Hospital, St. Mary Abbotts Hospital, Prince of Wales General Hospital, Tottenham. |
| BUNTING, J. B., M.B., B.CH. (CANTAB.), M.R.C.S. (Eng.), L.R.C.P. (Lond.), D.O. (Oxon), D.O.M.S. (Lond.). | Ophthalmologist, West Cornwall, Clinical Area. | Ophthalmic Practice Partnership and S.H.M.O., The London Hospital. |
| CREWE, T. C., B.D.S. (New Zealand). | S.H.D.O., South Devon and East Cornwall Hospital, Plymouth. | Assistant in general practice, Plymouth. |

| *Name* | *Appointment* | *Formerly* |
|---|---|---|
| DORAN, H. J., M.B., B.CH., D.L.O. | Clinical Assistant in E.N.T. Surgery, Torquay Group of Hospitals. | (*Extension of temporary contract for one session.*) |
| McGHIE, J. A., M.B., CH.B., M.R.C.O.G. | Clinical Assistant in Venereology, Torquay. | — |
| CHATURVEDI, R. C., M.B., B.S. (India) 1944, D.O. (Oxford). | Ophthalmologist (S.H.M.O.), Devon and Exeter Clinical Area. | Locum S.H.M.O., Mid-Glamorgan, H.M.C. |
| NORRIE, D. M., M.B., CH.B. (St. Andrews), D.A. | Registrar in Anaesthetics, Plymouth. | Senior House Officer (Anaesthetics), Plymouth. |

